# Association Between Medication Adherence and Oxidative Stress in Patients With First-Episode Mania

**DOI:** 10.3389/fpsyt.2019.00162

**Published:** 2019-03-26

**Authors:** Saínza García, Susana Alberich, Karina S. MacDowell, Mónica Martínez-Cengotitabengoa, Purificación López, Iñaki Zorrilla, Juan Carlos Leza, Ana González-Pinto

**Affiliations:** ^1^Department of Psychiatry, University Hospital of Alava-Santiago, Vitoria, Spain; ^2^Centre for Biomedical Research Network on Mental Health (CIBERSAM), Madrid, Spain; ^3^School of Medicine, University of the Basque Country, Vitoria, Spain; ^4^Department of Pharmacology and Toxicology, Faculty of Medicine, Complutense University of Madrid, IUIN and IIS Hospital 12 de Octubre, Madrid, Spain; ^5^Psychobiology Department, National Distance Education University (UNED), Vitoria, Spain

**Keywords:** medication adherence, oxidative stress, antioxidant activity, bipolar disorder, antipsychotic agents

## Abstract

Poor adherence is a major problem in patients with manic episodes that impairs functionality and has unknown effects on oxidative stress. The objective of this study was to analyze the relationship between adherence to medication, severity of symptoms and oxidative stress in a sample of patients with a first episode of mania. A longitudinal, 6-month study was performed in 60 patients, who were classified as adherent and non-adherent to medication (mainly antipsychotics). Blood levels of oxidative stress parameters and expression of the antioxidant nuclear transcription factor NRF2 in mononuclear cells of peripheral blood were assessed at baseline and at the end of follow-up. In addition, clinical symptoms and functioning were evaluated. Linear multivariate regression was used to determine the relationship between adherence, oxidative stress, and clinical symptoms. Finally, 44 patients completed follow-up. The results of this study showed that at 6-month follow-up, adherence was significantly associated with better functioning and reduced clinical symptoms. Additionally, more severe symptoms were associated with increased levels of oxidative stress and antioxidant parameters. At study completion, non-adherents exhibited greater levels of antioxidants than adherent patients. In conclusion, poor adherence to medication is associated with a poorer prognosis in the medium term and causes increased antioxidant response.

## Introduction

Bipolar disorder (BD) is a chronic disease that causes greater disability than cancer. It is estimated that 57% of BD patients are partially or totally non-adherent to medication (1). This lack of adherence causes a worsening of symptoms and increases of the risk for relapse, which may be related to neurobiological disbalance (2–5).

The physiopathology of BD has been the subject of intensive research, with a special focus on the search for biomarkers (6–8) that enable early diagnosis and a more accurate prognosis (9). Several studies agree about the analysis of total antioxidant status (TAS), which considers the cumulative effect of all antioxidant compounds present in plasma (10, 11). TAS has been proven to be decreased in patients with a first episode of mania (FEM) compared to healthy subjects (12). This parameter, that reflects disbalance in oxidative stress, has been related to worse clinical status of patients (13). In this context, nuclear factor erythroid 2-related factor-2 (NRF2) activity has a relevant role. In brief, NRF2 transcription factor regulates the expression of genes encoding antioxidants and detoxification enzymes by binding to a specific DNA sequence known as ARE (Antioxidant Response Element), found in the promoter regions.

Evidence has been recently provided that medication for BD has antioxidant effects. Thus, lithium—the first-choice therapy for BD- (14) has neuroprotective effect that reduces oxidative damage (15). In relation to antipsychotic drugs—one of the most powerful drugs for acute manic episodes (16)–, there is evidence that risperidone has an antioxidant effect (17). In animal models, paliperidone has been observed to regulate endogenous antioxidant pathways by preventing a decrease in antioxidant levels (18) or inducing its increase (19).

All this considered, and given that poor adherence is related to a higher risk for relapse and a worse prognosis of mania (20), we hypothesized that poor adherence and oxidative stress may be associated.

The objective of this 6-month follow-up study was to investigate the relationship among drug therapy, adherence, oxidative stress, and clinical symptomatology in a sample of subjects with a FEM. Our hypothesis was that adherent patients would have a higher antioxidant capacity, which will be reflected in a better clinical status.

## Materials and Methods

### Subjects

The sample was composed of 60 patients with a FEM admitted to the Department of Psychiatry of Araba University Hospital, Spain. Inclusion criteria were: (1) patients with a first episode of pure or mixed mania according to DSM IV-TR criteria (SCID-I and II) (21), (2) age of 12–40 years at disease onset, and (3) being fluent in Spanish. Exclusion criteria were: (1) mental retardation (assessed by DSM-IV-TR), (2) history of cranial trauma with loss of consciousness, (3) disease that causes mental health problems, (4) pervasive developmental disorders (PDD) and (5) pregnancy or breastfeeding.

The study was approved by the local Ethics Committee. Informed consent was obtained from all patients in accordance with the Declaration of Helskinki II. In subjects < 18 years of age, informed consent was obtained from their legal tutors after subjects agreed to participate in the study.

### Design and Clinical Assessment

This prospective longitudinal study involved the collection of blood samples and the performance of clinical assessments at baseline and at 6 months. Clinical evaluation was carried out by psychiatrists using the following instruments: the Spanish version of the Positive and Negative Syndrome Scale (PANSS), which measures the severity of symptoms of psychosis (22); the Hamilton Rating Scale for Depression (HRSD) (23); Young Mania Rating Scale (YMRS) (24); the Global Assessment of Functioning (GAF) (25) and the Global Assessment of Functioning in Children (CGAS) (26)—when appropriate–, which assesses functioning and severity of symptoms; and the Clinical Global Impression rating scale (CGI) (27), which assesses the severity and overall improvement of symptoms. Adherence to medication at the end of follow-up was assessed using Morisky-Green scale (28), by which patients were classified as adherent or non-adherent. This self-reported scale includes four yes/no questions rated on a 0–4 scale, considering patients who obtain 0 points as adherent and as not adherent those who obtain a 1+ score (29).This classification was subsequently confirmed by the patient's treating physician.

### Sample Collection and Preparation

Venous blood samples (10 mL) were collected early in the morning (between 8:00 and 9:00) after fasting overnight, by the nursing staff in polypropylene EDTA-containing tubes. Fresh blood was stored at 4°C until processing about 1 h. later (see Supplementary Material for more details).

### Biochemical Measurements in Plasma

Nitrites (${\text{NO}}_{2}^{-}$) the stable metabolites of free radical nitric oxide, were measured by the Griess method (30). Levels of lipid peroxidation (TBARS) were determined by Thiobarbituric Acid Reactive Substances (TBARS) assay (ref. 10009055, Cayman Chemical Europe), and the level of TAS was determined using the Antioxidant Assay Kit (ref. 709001, Cayman Chemical Europe) (For more details about the experimental procedure see Supplementary Material).

### Biochemical Measurements in Peripheral Blood Mononuclear Cells (PBMCs)

The activity of NRF2 was measured in nuclear extracts using a commercially available NRF2 Transcription Factor Assay Kit (ref. 600590, Cayman Chemicals) following manufacturer's instructions. A procedure based on the modified Schreiber method was used for the preparation of nuclear extracts (31).

### Statistical Analysis

Once normality of sociodemographic and clinical data was confirmed, descriptive analysis was performed. Results were expressed as means ± SD and percentages.

Differences between categorical variables inter-groups were assessed by the Chi-squared test. For continuous variables, differences were analyzed using the Student's *t*-test for independent samples. Intra-group differences in oxidative stress during follow-up were assessed using analysis of variance (ANOVA) with repeated measures. Backward stepwise regression were used to determine the relationship between adherence to medication and oxidative stress and symptoms. The same model was used to assess the relationship between symptoms and oxidative stress in the entire sample and determine potential associations among oxidative stress parameters. As CGI score is an ordinal variable, ordinal regression analysis was performed to assess the relationship between adherence and CGI score.

Models were controlled for potential confounding factors; significant confounding variables were included in the final models. When a (significant) confounding variable was included in a model, the interaction of independent variables with these confounding variables was also included.

Data are expressed as beta coefficients with *p*-values and the corresponding 95% confidence interval. Statistical analysis was performed using SPSS v23.0. A *p*-value < 0.05 was considered statistically significant.

## Results

Forty-four of the 60 initial subjects completed follow-up and were classified either as adherent (*n* = 28) or non-adherent (*n* = 16). Patients lost to follow-up were excluded from the final sample (*n* = 16). The characteristics of the sample are displayed in Table 1. At 6 months, the use of cannabis was significantly higher among non-adherent patients, as compared to adherent patients (χ^2^ = 7.06, *p* < 0.05). No differences were observed in relation to the treatment administered.

**Table 1:** Sociodemographic characteristics and oxidative parameters in a FEM sample classified by adherence to medication.

**Sociodemographic characteristics**	**Total patients (*****N*** **=** **44)**		**Adherent patients (*****N*** **=** **28)**		**Non-adherent patients (*****N*** **=** **16)**	
Age in years, n (SD)	27.09 (7.24)		28.36 (6.8)		25.25 (7.85)	
Sex (M,/F), n (%)	71.1 /28.9		78.6 /21.4		58.8 / 41.2	
**COHABITANTS**							
Parents and siblings	51.2		46.2		56.2	
Relatives	4.7		7.6		0	
Alone	16.3		23.1		6.3	
Partner and children	14.0		15.4		12.5	
Roommates	11.5		7.7		18.7	
Institutionalized	2.3		0		6.3	
**SOCIOECONOMIC STATUS, (%)**							
1 (lowest)	7.1		11.5		0	
2	14.3		3.8		33.3	
3	45.2		46.2		40	
4	21.4		23.1		20	
5(highest)	11.9		15.4		6.7	
**OCCUPATION, (%)**							
Medical or disability leave	4.7		3.7		6.3	
Student	23.3		22.2		25	
Unemployed	18.6		7.4		37.5	
Active	53.5		66.7		31.3	
**Substance use (%)**	**Baseline**	**At 6 months**	**Baseline**	**At 6 months**	**Baseline**	**At 6 months**
Tobacco	27.3	20.9	28.6	25.9	25	12.5
Alcohol	59.1	32.6	60.7	33.3	56.3	31.3
Cannabis	18.2	7	10.7	0	31.3	18.8
Other substances	15.9	0	7.1	0	31.3	0
**TYPE OF MEDICATION, (%)**						
Atypical antipsychotic	77.7	51.1	85.7	51.9	68.8	56.3
Atypical antipsychotic and lithium	20	17.7	14.3	18.5	31.3	18.8
Lithium	0	11.1	0	14.8	0	6.3
Others	0	15.5	0	14.8	0	18.8
**OXIDATIVE STRESS PARAMETERS**						
TAS, mM (SD)	7.65 (1.02)	2.5 (1.83)	7.57 (1.12)	2.04 (0.89)	7.75 (0.90)	3.25 (2.64)
TBARS, uM (SD)	1.61 (0.75)	1.9 (1.04)	1.66 (0.74)	1.75 (0.97)	1.53 (0.80)	2.21 (1.14)
${\text{NO}}_{2}^{-}$, Um (SD)	19.4 (9.88)	38.5 (29.66)	19.61 (9.32)	36.72 (29.42)	18.98 (11.71)	41.67 (31.83)
NRF2, A.U (SD)	0.40 (0.17)	0.57 (0.35)	0.39 (0.21)	0.49 (0.25)	0.41 (0.13)	0.64 (0.42)

### Relationship Between Adherence to Medication and Clinical Symptoms

Adherence to medication at 6 months showed a significant direct relationship with functioning as assessed by GAF (*B* = 11.36, 95% CI: 4.51–18.22, *p* < 0.01; after adjusting for alcohol an cannabis consumption at baseline, cannabis consumption at 6 months, sex, and education) and an inverse relationship with symptoms as measured by total PANSS (*B* = −9.32, 95% CI: −15.49 to −3.14, *p* < 0.01; after adjusting for education and alcohol consumption at baseline), general PANSS (*B* = −4.41, 95% CI: −7.77 to −1.06, *p* < 0.05; after adjusting for education, alcohol consumption at baseline and cannabis consumption at 6 months), HRSD (*B* = −3.75, 95% CI: −6.94 to −0.56; *p* < 0.05) and CGI severity scale (OR = 4.64, 95% CI: −2.795 to −0.272, *p* < 0.05; after adjusting for education).

### Relationship Between Oxidative Stress and Clinical Symptomatology in the Sample During Follow-Up

At 6-month follow-up, HRSD yielded a significant positive relationship with TAS (*B* = 0.10, 95% CI: −0.002 to −0.21, *p* = 0.05; after adjusting for alcohol consumption at baseline), whereas the GAF scale demonstrated an inverse significant relationship with this antioxidant parameter (*B* = −0.57, 95% CI: −0.10 to −0.01, *p* < 0.05; after adjusting for alcohol and tobacco consumption at baseline). CGI severity scale also presents a positive relationship with TAS, although in this case it was not significant (*B* = 0.346, 95% CI: −0.06–0.76, *p* = 0.083). Furthermore, negative PANSS scores showed a significant direct relationship with ${\text{NO}}_{2}^{-}$ values (*B* = 1.86, 95% CI: 0.14–3.60, *p* < 0.05; after adjusting for alcohol consumption at baseline).

The association between antioxidant capacity and the level of oxidative stress is supported by the direct relationship between TBARS and TAS parameters (*B* = 0.82, 95% CI: 0.22–1.42, *p* < 0.01), where antioxidant levels increased in response to increased oxidative damage.

### Relationship Between Adherence to Medication and Oxidative Stress

**Figure 1:**
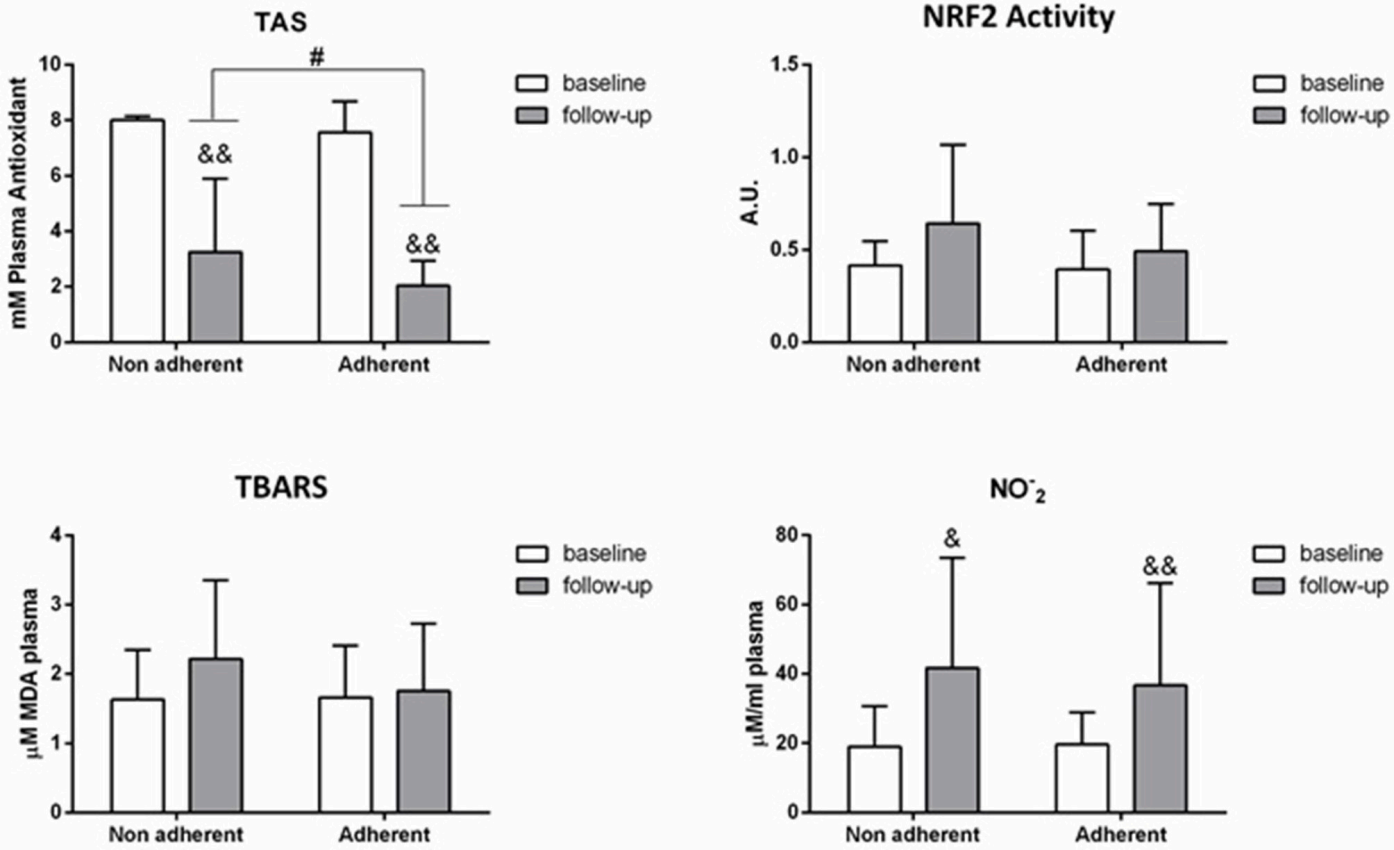
Mean differences ± SD in biomarkers between adherent and non adherent patients at 6-month follow-up. & *P* < 0.05, && *P* < 0.01 in the intra-group analyses between baseline and 6-months follow-up. # *P* < 0.05 between adherent and non adherent patients at 6-months follow-up.

Both, the adherent and non-adherent group exhibited a significant reduction in TAS (*F* = 178.99, *p* < 0.01; *F* = 29.14, *p* < 0.01, respectively) and a significant increase in ${\text{NO}}_{2}^{-}$ at 6-month follow-up (*F* = 8.44; *p* < 0.01; *F* = 5.42, *p* < 0.05). The non-adherent group presented a significantly lower decrease in TAS levels (*t* = −2.08, *p* < 0.05; Figure 1) and a non-significant rise of NRF2 activity and oxidative stress levels (TBARS, ${\text{NO}}_{2}^{-}$) compared to the adherent group at the end of follow-up.

There was a significant inverse relationship between adherence and TAS levels at 6- month follow-up, with an increased antioxidant capacity in patients with poor therapeutic compliance (*B* = −1.46; 95% CI: −2.60 to −0.32, *p* < 0.05; after adjusting for tobacco consumption at 6 months).

## Discussion

In the light of the results obtained, efforts should be made to ensure treatment adherence in BD patients. Treatment adherence results in an improvement of symptoms, which has been associated with the reduction of oxidative stress. Therefore, as non-adherent patients have more severe symptoms, their oxidative stress increases and their antioxidant capacity augments as an adaptive response of the body. This phenomenon is demonstrated by the direct relationship observed in our study between TBARS and TAS at 6 months in the entire sample. A previous study (17) in 51 patients with a first episode of psychosis showed that levels of oxidative stress diminished following the administration of risperidone. We obtained similar results for FEM patients who underwent longer follow-up and found that adherence is crucial when assessing response to treatment based on clinical and biological parameters such as oxidation. Non-adherence to medication is especially important in patients with bipolar disorder (1). Conversely to our initial hypothesis, TAS levels were higher in non-adherent patients as compared to adherent patients at the end of follow-up. We hypothesize that this phenomenon may be associated with small, non-significant increases in oxidative stress, thereby supporting the relationship between TBARS and TAS. The patients who did not take correctly their prescriptions had worse clinical outcomes, which is consistent with previous studies (4, 5). Additionally, non-adherent patients maintained higher antioxidant levels in the long term. To our knowledge, this is the first study that shows the relationship between lack of adherence and higher TAS in the long term. Increased oxidative stress (manifested as higher levels of TAS) probably triggers a complex sequence of events that lead to the migration of NRF2 into the nucleus, where it binds to ARE, thereby increasing the antioxidant capacity of the patient (32). In our sample there was a non-significant increase in NRF2. As shown by other authors, to maintain homeostatic balance, NRF2 remains inactive in the cytoplasm until oxidative stress conditions emerge (33, 34).

Conflicting results have been obtained in a variety of studies on the antioxidant effect of treatments based on typical and atypical antipsychotics and lithium, and how they regulate oxidative stress in preclinical studies (18, 19, 35–39). In our study, an increase in oxidative stress levels and a decrease in antioxidant response were noted in the two groups at 6 months, regardless of the medication administered. As shown in previous studies, oxidative stress diminished in the patients who took their prescriptions correctly (17). Surprisingly, oxidative stress also decreased at 6 months in non-adherent patients, something that had not been ever reported, to our knowledge. Yet, a direct relationship was found between clinical symptoms and oxidative stress and antioxidant capacity at 6 months. This has been shown in patients with a first episode of psychosis after 6-month follow (40), and in patients with early psychosis after 2-year follow- up (13). However, this association was not observed in other studies based on 11-week follow-up (17), or 6 weeks follow-up (41). Antipsychotic therapy may have positive effects on oxidation, as it may have intrinsic antioxidant properties in the short term not related to symptoms. However, after 6 months, to achieve oxidative balance in non-adherent patients, the body reduces oxidation by increasing TAS. The reason for this phenomenon may be that adherent patients reach oxidative balance faster than non-adherent patients. This hypothesis is supported by the results of Noto et al., which showed that oxidative balance at 11 weeks was not related to symptom improvement (17). In contrast, our results after 6-month follow-up reflect an association between antioxidant status and clinical severity. More specifically, antioxidant status was found to be related to depression, negative symptoms, and functional outcome.

This study has some limitations. First, the high number of patients lost to follow-up at 6 months. This may be due to the characteristics of patients with a FEM, whose level of awareness is generally low or nonexistent. Second, we could not assess the neuroprotective effect of lithium due to the small proportion of patients who received lithium therapy. Third, only indirect methods based on interviews and reports from clinicians were used to assess adherence. However, most studies use indirect methods to assess adherence as compared to direct methods (77 vs. 23%, respectively) (42).

In sum, contrary to what has been previously demonstrated in the short term, oxidative stress and antioxidant capacity might not be directly modulated by medication at 6 months, but indirectly by symptoms. Thus, adherence to medication may have an indirect mediation effect on oxidative stress. The mechanism is as follows: with the medication, clinical status improves, which results in a lower activation of the antioxidant system. These 6-month follow-up results shed light on the role of oxidative status in psychiatric diseases, its relationship with clinical status, and the effects of medication on clinical status and antioxidant capacity. Larger, long-term studies with repeated measures at short and long term follow ups are needed to validate the results of this study and confirm the importance of good adherence to medication.

## Data Availability

The datasets for this manuscript are not publicly available because databases contain confidential information about patients and healthy subjects. Requests to access the datasets should be directed to SG, sainza.garciafernandez@osakidetza.eus.

## Ethics Statement

This study was carried out in accordance with the recommendations of the Ethics Committee of Araba University Hospital with written informed consent from all subjects. All subjects gave written informed consent in accordance with the Declaration of Helsinki. The protocol was approved by the Ethics Committee of Araba University Hospital.

## Author Contributions

AG-P and JL designed the current study. All authors contributed to the acquisition of the data. SG, PL, and IZ managed the literature searches. KM and SG performed biochemical determinations in plasma and cells. SG and SA undertook the statistical analysis. SG, MM-C, AG-P, and JL wrote the first version of the manuscript and all authors contributed to and have approved the final version.

### Conflict of Interest Statement

IZ has received grants and served as consultant, advisor or CME speaker for the following entities: Janssen-Cilag, Ferrer, Johnson & Johnson, Lundbeck, Pfizer, Sanofi-Aventis, the Spanish Ministry of Science and Innovation (CIBERSAM), the Ministry of Science (Carlos III Institute), the Basque Government and the Stanley Medical Research Institute. AG-P has received grants and served as consultant, advisor or CME speaker for the following entities: Almirall, AstraZeneca, Bristol-Myers Squibb, Cephalon, Eli Lilly, Glaxo-Smith-Kline, Janssen-Cilag, Ferrer, Johnson & Johnson, Lundbeck, Merck, Otsuka, Pfizer, Sanofi-Aventis, Servier, Shering-Plough, Solvay, the Spanish Ministry of Science and Innovation (CIBERSAM), the Ministry of Science (Carlos III Institute), the Basque Government, the Stanley Medical Research Institute, and Wyeth. The remaining authors declare that the research was conducted in the absence of any commercial or financial relationships that could be construed as a potential conflict of interest.
