# Supplementary material for: Association Between Medication Adherence and Oxidative Stress in Patients With First-Episode Mania
Source: Front Psychiatry. 2019 Mar 26;10:162. doi: 10.3389/fpsyt.2019.00162 (PMC6445053; doi:10.3389/fpsyt.2019.00162)
Supplement: Supplementary file 1 [file Data_Sheet_1.docx]

Supplementary Material

Association between medication adherence and oxidative stress in patients with first-episode mania

Saínza García, Susana Alberich, Karina S. MacDowell, Mónica Martínez-Cengotitabengoa, Purificación López, Iñaki Zorrilla, Juan Carlos Leza, Ana González-Pinto^*^

*** Correspondence:** Ana González-Pinto: Anamaria.gonzalez-pintoarrillaga@osakidetza.eus

**Supplementary Methods**

*Further information about sample collection, preparation and biochemical measurement*

Venous blood samples (10 mL) were collected early in the morning (between 8:00 and 9:00) by the nursing staff in in polypropylene EDTA-containing tubes. Fresh blood was stored at 4ºC until processing about 1 h. later. Blood was centrifuged (652g x 10 min, 4°C); the resulting plasma was carefully collected and stored at -80°C until use.

The resulting supernatant was diluted 1:1 in culture medium (Roswell Park Memorial Institute [RPMI] 1640, GIBCO). In a tube, 0.65 mL of Ficoll-Paque (GE Healthcare) was used per 1 mL of blood / RPMI mixture and layered slowly over Ficoll-Paque solution to create a concentration gradient of 800 g for 40 min. at room temperature. Peripheral blood mononuclear cells (PBMC) layer together with RPMI were absorbed and diluted with an equal volume of RPMI. The mixture was centrifuged again at room temperature at 1800g for 15 min. The supernatant was removed and the PBMCs pellet was resuspended in 1mL of RPMI. The mixture was centrifuged at room temperature at 1800g rpm for 10 min. After the supernatant was removed, the pellet was stored at -80ºC until analysis.

*Biochemical measurements in plasma*

Nitrites (NO^−^_2_). The stable metabolites of free radical nitric oxide were measured in plasma by the Griess method (Green et al., 1982). Briefly, in an acidic solution with 1% sulphanilamide and 0.1% N-(2-napthyl) ethylenediamine dihydrochloride (NEDA), nitrites convert into a pink compound that is measured by spectrophotometry calculated at 540nm in a microplate reader (Synergy 2, BioTek)

Lipid Peroxidation (TBARS). This byproduct of lipid peroxidation was determined in plasma by Thiobarbituric Acid Reactive Substances (TBARS) assay (ref. 10009055, Cayman Chemical Europe), based on the reaction of malondialdehyde and thiobarbituric acid under high temperature (95ºC) and acidic conditions measured colorimetrically at 530 nm (Synergy 2, BioTek).

Total Antioxidant Status (TAS). Was determined in plasma by Antioxidant Assay Kit (ref. 709001, Cayman Chemical Europe) based on the ability of antioxidants presents in the sample to inhibit the oxidation of ABTS (2, 2’-azino-bis (3-ethylbenzthiazoline-6-sulphonicacid)), which is monitored by reading absorbance at 750 nm.
